# Supplementary material for: Access to Healthcare for Migrant Patients in Europe: Healthcare Discrimination and Translation Services
Source: Int J Environ Res Public Health. 2021 Jul 26;18(15):7901. doi: 10.3390/ijerph18157901 (PMC8345338; doi:10.3390/ijerph18157901)
Supplement: Supplementary file 1 [file ijerph-18-07901-s001.zip › ijerph-1211657-supplementary.pdf]

Supplementary Table S1. DMS questionnaire. Each question of the DMS scale scores from 1: Never true to 5: Always true.

	Question
Q1	You are treated with less courtesy than other people.
Q2	You are treated with less respect than other people.
Q3	You receive poorer service than others.
Q4	A doctor or nurse acts as if he or she thinks you are not smart.
Q5	A doctor or nurse acts as if he or she is afraid of you.
Q6	A doctor or nurse acts as if he or she is better than you.
Q7	You feel like a doctor or nurse is not listening to what you were sayin.

Supplementary Table S2. Pearson's correlation and Cronbach's diagonal results for each question with total DMS scale. Migrants and refugees from Germany, France and Malta were excluded from the analysis.

Pearson correlations*	*DMS scale*	*Q1*	*Q2*	*Q3*	*Q4*	*Q5*	*Q6*	*Q7*
DMS scale	1.0000							
Q1	0.8171	1.0000						
Q2	0.8576	0.7718	1.0000					
Q3	0.8190	0.6490	0.6932	1.0000				
Q4	0.8431	0.5707	0.6424	0.6170	1.0000			
Q5	0.7944	0.5338	0.6159	0.5852	0.7043	1.0000		
Q6	0.8701	0.6459	0.6911	0.6486	0.7254	0.6623	1.0000	
Q7	0.8261	0.6027	0.6132	0.5831	0.6709	0.6109	0.7234	1.000
Cronbach's a	0.9273	0.9185	0.9128	0.9184	0.915	0.9198	0.9112	0.9178

**p<0.001 in all correlations*

Supplementary Table S3. Pearson's correlation and Cronbach's diagonal results for each question with total Mental Health Score. Migrants and refugees from Germany, France and Malta were excluded from the analysis.

	Pearson correlations*	***Cronbach's a***
Have you been a very nervous person?	0.6319	0.7448
Have you felt so down in the dumps that nothing could cheer you up?	0.7872	0.6643
Have you felt calm and peaceful?	-0.6950	0.7193
Have you felt downhearted and blue?	0.7826	0.6679
Have you been a happy person?	-0.6548	0.7361
Total Mental Health Score	1.0000	0.7521

**p<0.001 in all correlations*

Supplementary Table S4. Characteristics of the total sample of migrants and refugees (N=1407).

	N (%)
Country of interview	
Austria	126 (8.96)
Germany	11 (0.78)
Bulgaria	226 (16.06)
Cyprus	110 (7.82)
France	64 (4.55)
Greece	255 (18.12)
Italy	271 (19.26)
Malta	38 (2.7)

Spain	202 (14.36)
Sweden	104 (7.39)
Country of origin	
Afghanistan	211 (15.18)
Iran	48 (3.45)
Iraq	127 (9.14)
Nigeria	118 (8.49)
Syria	294 (21.15)
Other	592 (42.59)
Gender (Males)	889 (63.45)
Having at least one child	575 (50.26)
Asylum (Yes)	358 (28.94)
Other kind of permission (Yes)	619 (46.37)
Speaking country of interview language (Yes)	484 (40.27)
Comorbidity	
Not having a disease or chronic condition	713 (50.68)
Having one disease or chronic condition	334 (23.740
Having at least two diseases or chronic conditions	360 (25.59)
	Mean ± Standard deviation
Age (years)	31.98±11.05
Education (years)	9.1±5

Supplementary Table S5. Communication skills by country of origin and country of interview. Migrants without DMS score (missing values) were excluded from the analysis.

	Not speaking country of interview language	Speaking country of interview language
Country of origin (%)***		
Afghanistan	27 (79.41)	7 (20.59)
Iran	14 (70)	6 (30)
Iraq	58 (84.06)	11 (15.94)
Nigeria	25 (50)	25 (50)
Syria	144 (87.8)	20 (12.2)
Other	105 (34.54)	199 (65.46)
Country of interview (%)***		
Austria	64 (70.33)	27 (29.67)
Bulgaria	48 (96)	2 (4)
Cyprus	61 (98.39)	1 (1.61)
Greece	119 (95.2)	6 (4.8)
Italy	49 (44.95)	60 (55.05)
Spain	10 (6.25)	150 (93.75)
Sweden	25 (52.08)	23 (47.92)

*** $p<0.001$, ** $p<0.05$, * $p<0.1$
